# Spontaneous Intestinal Perforation in Neonates

**Published:** 2015-04-01

**Authors:** Charu Tiwari, Gursev Sandlas, Shalika Jayaswal, Hemanshi Shah

**Affiliations:** Department of Paediatric Surgery, TNMC and BYL Nair Hospital, Mumbai, Maharashtra, India

**Keywords:** Perforation, Spontaneous, Neonatal, NEC, Hirschsprung’s disease

## Abstract

Background: The term Spontaneous Intestinal Perforation (SIP) suggests a perforation in the gastrointestinal tract of a newborn with no demonstrable cause.

Methods: Four neonates presenting with spontaneous bowel perforation were analyzed with respect to clinical presentation, management and outcome.

Results: The mean age at presentation was 11.4 days. There were three males and one female. One of the neonates was preterm, very low birth weight and the other three were full term. Two neonates underwent emergency exploratory laparotomy and two were initially managed by peritoneal drainage in view of poor general condition; one of them improved and did not require further operative intervention. The preterm very low birth weight neonate was stabilized and explored after 48 hours. Intra-operatively, two of them had two ileal perforations each which required ileostomy; one had single perforation in the transverse colon which was primarily repaired. All four had an uneventful recovery.

Conclusion: SIP is a distinct clinical entity and has better outcome than neonates with intestinal perforation secondary to Necrotizing Enterocolitis (NEC).

## INTRODUCTION

Neonatal bowel perforation may have varied etiology - spontaneous, secondary to NEC, and mechanical obstruction etc. [1]. The term SIP suggests a perforation in the gastrointestinal tract of a newborn with no demonstrable cause that is typically found in the terminal ileum [1]. Though seen frequently in pre-term newborns with very low birth weight (VLBW) and extremely low birth weight (ELBW), only a few cases have been described in full-term newborns [1-4]. The etiology and pathogenesis of the disease is unknown and multiple theories have been proposed, but, none has been proven. Conditions associated with fetal or neonatal hypoxia are important antecedents for this emerging distinct entity [1]. It is a separate clinical entity from NEC and this differentiation is important because of management and outcome considerations.

## MATERIALS AND METHODS

Four neonates presenting with acute abdomen as a result of spontaneous bowel perforation were analyzed with respect to clinical presentation, management and outcome. 

They had no associated bowel disease and no clinical features of necrotizing enterocolitis, Hirschsprung’s disease or anorectal malformations as per the history, clinical presentation, operative findings, and histopathology.

## RESULTS

**Demographic Details:** The mean age at presentation was 11.4 days. One of them was preterm VLBW twin baby and the rest three were full term neonates. Three were male and one was female. (Table 1) 

**Figure:**
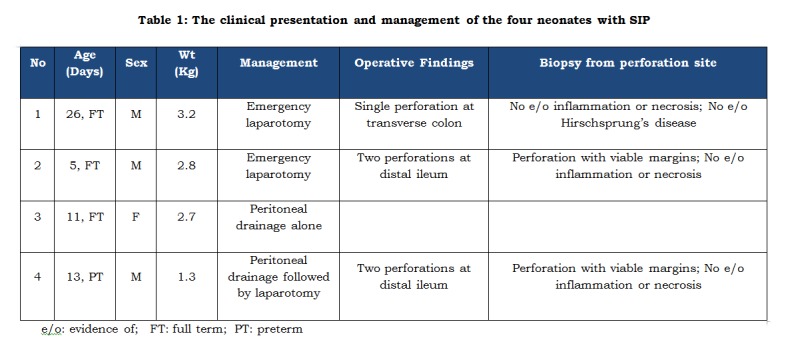
Table 1: The clinical presentation and management of the four neonates with SIP

**Clinical Presentation:** Abdominal distention and vomiting for 3 to 4 days were the presenting symptoms in all the patients. The preterm VLBW neonate had associated complaint of constipation. The three full-term neonates had normal delivery and good birth weight. The perinatal history was uneventful for the three full term neonates; but the preterm neonate had history of mechanical ventilation for four days and surfactant therapy for hyaline membrane disease was administered, in a private hospital. On examination, all had abdominal distention; the preterm neonate also had erythema of the abdominal wall.

**Management:** All the four neonates had pneumoperitoneum on X-ray abdomen erect posture. The two full term male neonates were emergently explored. One was managed by peritoneal drainage alone. The preterm neonate was in sepsis with huge abdominal distention and erythema with low platelet counts; so he was initially managed by peritoneal drainage and was taken for exploration after stabilization. One had a single perforation at transverse colon (Fig. 1) which was primarily repaired as the history and intra-operative appearance of bowel was not suggestive of Hirschsprung’s disease and the perforation was very small; two neonates had two perforations each in the distal ileum managed by resection of the perforated segment, ileostomy, and mucous fistula formation (their bowel condition was also not suggestive of Hirschsprung’s disease or NEC). Biopsy was taken only from the site of perforation and no distal biopsies were taken. 

**Figure:**
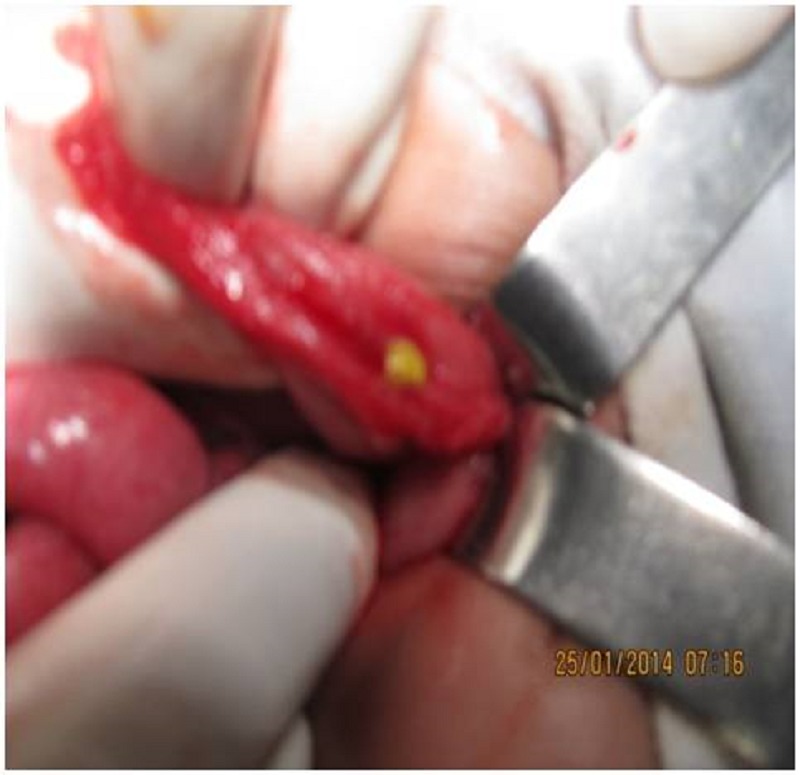
Figure 1: Intra-operative image of the single perforation in the transverse colon.

The patient managed by primary peritoneal drainage was a full term female neonate who responded well to initial resuscitation and peritoneal drainage. The drain output included air and bile initially which stopped within 4 to 5 days after which she was started oral feeds. 

None of the three operated neonates had features of any intrinsic bowel disease, necrosis or gangrene. The whole bowel was healthy apart from the perforation site.

**Outcome:**

All the three full term neonates had a rapid recovery and were discharged within 7 days and are doing well on follow-up. The preterm neonate had to be kept on ventilator and ionotropic support for three days. He was then gradually weaned off ventilator and started on Ryle’s Tube feeds. He was gaining weight and discharged for personal reasons on post-operative day 11.

The histopathology reports (Frozen Section couldn’t be done as it is not available in emergency at our institute) did not reveal Hirschsprung’s disease or NEC.

## DISCUSSION

Pneumoperitoneum is usually an indication of perforated hollow viscera and requires urgent surgical intervention [5]. NEC is the most common cause of pneumoperitoneum in premature neonates [6]. Stress, hypoxia, or shock may lead to regional hypo-perfusion and transient intestinal ischemia resulting in SIP. Premature rupture of membranes, lower Apgar scores, and cardiovascular resuscitation in the perinatal period may prone the neonate to SIP. The terminal ileum is mostly affected site however SIP is also reported in the transverse and descending colons [7]. SIP is the second most common cause of neonatal intestinal perforation [8] and has been very well documented in the low-birth-weight neonates [9, 10]. Its incidence is 1.1% in VLBW and 7.4% in ELBW neonates [4 ]. Only a few cases have been described in full-term neonates [1]. In our study, we had three full-term neonates with SIP and only one pre-term neonate. All the three full-term neonates had good birth weight and only the pre-term neonate had VLBW. This finding is in contrast with other reports. [9-11]. 

Closed peritoneal drainage has been suggested as a primary or definitive procedure [12-15 ]. We had two different scenarios where peritoneal drainage was done primarily- one was a full term female neonate who responded to initial resuscitative measures and had no peritoneal inflammatory signs; this neonate settled well without a laparotomy. The other was a pre-term VLBW neonate in whom the peritoneal drainage was done to gain time for stabilization. He was explored two days later. Thus, peritoneal drainage can serve either as a definitive management or as a temporary measure to buy time for stabilizing the patient. Nevertheless, few authors also reported conservative management without even primary peritoneal drainage in stable patients with no peritoneal signs [7]. In majority of cases the SIP is considered single perforation involving particularly the terminal ileum; however, it may involve any part of alimentary tract. Only few cases of multiple spontaneous intestinal perforations have been encountered [16].

All these four cases were assumed to be SIP because:

1) X-Ray: No evidence of portal venous gas or pneumatosis intestinalis.

2) Intra-operatively: Healthy bowel apart from the perforation site and no evidence of distal obstruction.

3) At Histopathology: No evidence of NEC.

4) Outcome: Good.

## Conclusion

SIP is a distinct clinical entity and unlike NEC, has no long-term gastrointestinal sequel. Primary peritoneal drainage can serve as primary management for a very sick neonate to gain time for stabilization or it alone can treat the patient. Nevertheless, a very minute sample size is inadequate to draw any concrete conclusions; however, distinction between SIP and NEC is important for management and outcome considerations.

## Footnotes

**Source of Support:** None

**Conflict of Interest:** None

